# Renal Involvement in Indolent and Aggressive B-Cell Neoplasms: A Comparative Study of Chronic Lymphocytic Leukemia/Small Lymphocytic Lymphoma and Diffuse Large B-Cell Lymphoma

**DOI:** 10.3390/curroncol33070420

**Published:** 2026-07-14

**Authors:** Yiming Zhao, Bingjie Wang, Huihui Liu, Xiaoying Yang, Zhizhen Lai, Bo Tang, Weiwei Xie, Hongtao Ling, Shuanglian Xie, Shujing Guo, Xiaojuan Yu, Yujun Dong

**Affiliations:** 1 Department of Hematology, Peking University First Hospital, Beijing 100032, China; 2 Department of Nephrology, Peking University First Hospital, Beijing 100032, China

**Keywords:** chronic lymphocytic leukemia/small lymphocytic lymphoma, diffuse large B-cell lymphoma, renal involvement, kidney biopsy, hematologic response, high-grade B-cell lymphoma

## Abstract

Renal involvement is an uncommon but clinically important manifestation of B-cell neoplasms, and direct comparisons between indolent and aggressive entities remain limited. This single-center retrospective biopsy-confirmed and tissue-selected study compared 28 biopsy-confirmed patients with chronic lymphocytic leukemia/small lymphocytic lymphoma (CLL/SLL) or diffuse large B-cell lymphoma/high-grade B-cell lymphoma (DLBCL/HGBL), with 14 patients in each group, treated at Peking University First Hospital between June 2010 and June 2025. The aggressive comparator group included 13 DLBCL cases and one case annotated as HGBL with MYC and BCL2 rearrangements. Clinical features, timing of renal involvement recognition, dominant clinical entry points, pathological patterns, treatment strategies, and hematologic and renal responses were analyzed. CLL/SLL was associated with higher white blood cell and absolute lymphocyte counts, whereas DLBCL/HGBL showed higher lactate dehydrogenase and β2-microglobulin levels. The interval to renal involvement recognition was longer in CLL/SLL than in DLBCL (24.00 vs. 2.00 months, *p* = 0.007). At renal involvement recognition or biopsy, 24 h urinary protein excretion was nominally higher in the CLL/SLL group than in the DLBCL/HGBL group (3.90 vs. 1.58 g/24 h). CLL/SLL more often presented with proteinuria/edema, hematuria, or renal dysfunction and showed heterogeneous infiltrative lesions with concurrent glomerular or vascular involvement. DLBCL/HGBL more frequently presented with flank pain or renal mass-related manifestations and was dominated by direct infiltrative or mass-forming lesions. Treatment patterns differed markedly, whereas no significant difference in the distribution of renal responses was detected; however, this comparison was underpowered and should be interpreted descriptively. In this biopsy-confirmed, tissue-selected cohort, CLL/SLL and DLBCL/HGBL showed different observed patterns of renal involvement recognition, tissue acquisition, and renal pathological presentation. These findings support tissue-based evaluation of renal abnormalities in B-cell neoplasms but should be interpreted as descriptive and hypothesis-generating in view of the small sample size and the influence of diagnostic and biopsy pathways.

## Simple Summary

Kidney problems can occur in patients with different types of B-cell blood cancers, but their recognition and tissue findings may differ across diagnostic pathways. In this single-center retrospective, tissue-selected study, we compared patients with chronic lymphocytic leukemia/small lymphocytic lymphoma and patients with diffuse large B-cell lymphoma/high-grade B-cell lymphoma who had kidney involvement confirmed by tissue biopsy. We found that kidney involvement was usually recognized later and showed more mixed kidney tissue changes in chronic lymphocytic leukemia/small lymphocytic lymphoma, whereas the DLBCL/HGBL group was more often associated with kidney masses or direct tumor infiltration over a shorter period. Because all patients were selected on the basis of available tissue, these findings should be interpreted as descriptive patterns rather than proof that the two disease groups intrinsically affect the kidney in different ways. These findings support careful tissue-based evaluation of kidney abnormalities in B-cell blood cancers within the appropriate clinical and diagnostic context.

## 1. Introduction

Chronic lymphocytic leukemia/small lymphocytic lymphoma (CLL/SLL) and aggressive B-cell lymphomas, including diffuse large B-cell lymphoma (DLBCL) and high-grade B-cell lymphoma (HGBL), are clinically relevant B-cell neoplasms, but they differ substantially in disease biology, clinical tempo, and therapeutic framework. CLL/SLL generally follows a relatively indolent course and is commonly managed according to iwCLL criteria [1], whereas DLBCL/HGBL represents an aggressive lymphoma context [2] for which staging and response assessment are usually based on the Lugano classification [3]. In clinical hematology, renal involvement should not be regarded merely as an isolated organ complication, because it may reflect extranodal disease, immune dysregulation, monoclonal protein-related injury, or treatment-associated toxicity, thereby influencing overall disease evaluation and management [4].

Previous studies have shown that renal involvement in CLL/SLL is markedly heterogeneous. Clinically, it may present with proteinuria, nephrotic syndrome, impaired renal function, or acute kidney injury, while pathologically it may reflect leukemic infiltration, immune-mediated glomerular lesions, or monoclonal protein-related damage [5,6,7]. By contrast, renal involvement in DLBCL/HGBL or broader non-Hodgkin lymphoma cohorts is more often associated with aggressive extranodal disease and may manifest as acute renal dysfunction, parenchymal infiltration, renal enlargement, or mass-forming lesions [8,9]. More recent clinicopathological studies of B-cell lymphoproliferative disorders have further suggested that the clinical entry point, histologic pattern, and outcome of renal involvement are closely related to the underlying hematologic malignancy [10,11]. However, direct comparative data between CLL/SLL and DLBCL/HGBL with renal involvement remain limited, particularly studies integrating clinical presentation, renal histopathology, and treatment-related outcomes within the same analytical framework. Recent reports published within the past three years have continued to illustrate that CLL/SLL may rarely present with biopsy-confirmed acute kidney injury, whereas DLBCL/HGBL and renal lymphoma may present with renal masses, obstructive or infiltrative renal involvement, or glomerular manifestations [12,13,14,15].

Therefore, in the present study, we included biopsy-confirmed patients with renal involvement in the setting of CLL/SLL or DLBCL/HGBL and systematically compared the tempo of renal involvement recognition, clinical entry point, pathological expression, treatment strategies, and treatment-related outcomes within a single cohort using uniform study definitions. In contrast to previous kidney biopsy series of B-cell lymphoproliferative disorders, this study specifically integrated timing of renal involvement recognition, dominant clinical presentation, tissue acquisition route, structured pathological categories, and disease-specific hematologic response criteria in the same analytical framework. The aim was to describe and compare the observed clinical, pathological, and response patterns of renal involvement in these two biopsy-confirmed, tissue-selected groups, while recognizing that this retrospective design cannot establish intrinsic renal tropism. CLL/SLL and DLBCL/HGBL were therefore compared not because they are biologically equivalent or should be managed together, but because they represent clinically relevant indolent and aggressive B-cell neoplasms that may both require hematology–nephrology evaluation and tissue confirmation when renal abnormalities occur.

## 2. Materials and Methods

### 2.1. Study Design and Patients

**Figure 1:**
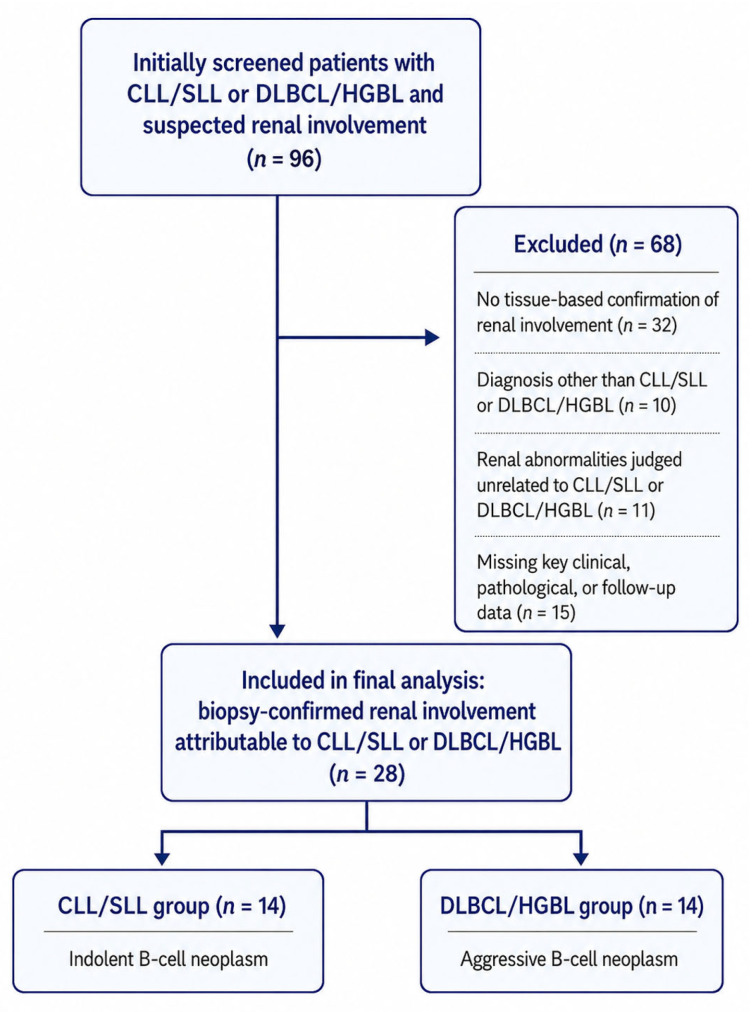
Patient selection flowchart. Patients were screened from the renal pathology archive and hematology records between June 2010 and June 2025. A total of 96 patients with B-cell neoplasms and suspected renal involvement were initially screened, and 28 biopsy-confirmed patients with renal involvement attributable to CLL/SLL or DLBCL/HGBL were included in the final analysis. Among the 68 excluded patients, 33 had CLL/SLL, 25 had DLBCL/HGBL, and 10 had other B-cell neoplasms; the disease-group breakdown by exclusion reason is provided in Supplementary Table S4.

This was a single-center retrospective study conducted at Peking University First Hospital. Patients with biopsy-confirmed renal involvement in the setting of chronic lymphocytic leukemia/small lymphocytic lymphoma (CLL/SLL) or diffuse large B-cell lymphoma/high-grade B-cell lymphoma (DLBCL/HGBL) were identified from the renal pathology archive and hematology records between June 2010 and June 2025. CLL and SLL were analyzed together as one disease entity according to the current iwCLL framework, whereas all DLBCL/HGBL cases required pathological confirmation [1,3]. The patient selection process is shown in Figure 1. This study was approved by the Ethics Committee of Peking University First Hospital (approval No. 2021yan323). The ethics approval and subsequent continuing reviews covered the retrospective review of archival clinical and pathological data from June 2010 to June 2025. Because of the retrospective nature of the study and the use of de-identified data, the requirement for individual informed consent was waived by the Ethics Committee. The study was conducted in accordance with the Declaration of Helsinki and institutional ethical requirements. The aggressive B-cell neoplasm group included 13 DLBCL cases and one case annotated as high-grade B-cell lymphoma (HGBL) with MYC and BCL2 rearrangements according to WHO HAEM5 and ICC 2022 criteria [16,17]. This case retained its original case identifier (DLBCL-12) for traceability and was retained in the aggressive comparator group, which is referred to as the DLBCL/HGBL group where appropriate.

Eligible cases met all of the following criteria: (1) a definite diagnosis of the underlying hematologic malignancy; (2) objective evidence of renal involvement, including urinary abnormality, renal dysfunction, or imaging-detected renal or renal hilar lesion; (3) available tissue-based evidence allowing pathological characterization of renal involvement, including native kidney biopsy or image-guided biopsy of a renal mass or hilar lesion; and (4) relatively complete clinical, pathological, treatment, and follow-up data. Patients were excluded if they had (1) a diagnosis other than CLL/SLL or DLBCL/HGBL, (2) no tissue-based confirmation of renal involvement, (3) renal abnormalities judged unrelated to CLL/SLL or DLBCL/HGBL, or (4) missing key clinical, pathological, treatment, or follow-up data precluding comparative analysis.

### 2.2. Definition of Renal Involvement and Data Collection

To accommodate the heterogeneous patterns of kidney involvement in B-cell lymphoproliferative disorders, renal involvement was used as an umbrella term referring to biopsy-supported renal or renal hilar involvement in the setting of CLL/SLL or DLBCL/HGBL. Functional or urinary renal involvement was defined by at least one of the following: proteinuria, hematuria, elevated serum creatinine/renal dysfunction, nephrotic-range proteinuria or nephrotic syndrome, or dialysis requirement. Proteinuria was defined as documented abnormal urinary protein excretion, and nephrotic-range proteinuria was defined as 24 h urinary protein excretion > 3.5 g/24 h. Hematuria was defined as documented microscopic or gross hematuria in urinalysis or medical records. Renal dysfunction was defined as elevated serum creatinine above the institutional reference range, reduced estimated glomerular filtration rate (eGFR < 60 mL/min/1.73 m^2^), or dialysis requirement. Imaging-defined renal involvement was defined as renal parenchymal, hilar, obstructive, or mass-forming lesions subsequently supported by tissue-based evidence of renal involvement. This broad definition was used to capture the main clinical routes through which renal involvement was recognized.

Baseline variables collected at the time of diagnosis of the underlying hematologic malignancy included age, sex, ECOG performance status, hepatosplenomegaly, white blood cell count, absolute lymphocyte count, hemoglobin, platelet count, lactate dehydrogenase, β2-microglobulin, albumin, 24 h urinary protein excretion, serum creatinine, immunofixation electrophoresis, and cryoglobulin testing (Table 1). Available disease-specific variables were also collected, including IGHV mutation status, TP53 mutation, del(17p), del(11q), del(13q), trisomy 12, and monoclonal component information for CLL/SLL, and Richter transformation status, Ann Arbor stage, IPI score, extranodal sites, cell-of-origin/Hans classification, TP53 abnormality, MYC/BCL2/BCL6 expression, MYC/BCL2 double expression, and MYC/BCL2/BCL6 FISH rearrangement results for DLBCL/HGBL when available (Supplementary Table S1). IHC-defined MYC/BCL2 double-expression status and FISH-defined double-hit status were treated as distinct constructs. Prior systemic therapy before renal involvement recognition was recorded when available. The interval from hematologic malignancy diagnosis to recognition of renal involvement was recorded. Because the renal-related laboratory variables in the baseline analysis reflected the disease context at hematologic diagnosis, additional renal parameters at renal involvement recognition or biopsy were separately summarized, including serum creatinine, eGFR, 24 h urinary protein excretion, hematuria, nephrotic syndrome or nephrotic-range proteinuria, dialysis requirement, renal mass/hilar lesion or obstruction on imaging, and tissue acquisition approach (Table 2).

Dominant renal-related presenting manifestations were assigned according to the principal presenting feature documented in the medical records and categorized as proteinuria/edema-predominant, hematuria-predominant, renal insufficiency/elevated creatinine-predominant, renal mass/flank pain-predominant, or lymphoma-related symptoms/incidental abnormality-predominant. These categories were used to describe the main clinical entry point to renal involvement recognition rather than to define an internationally standardized classification.

### 2.3. Renal Tissue Acquisition and Pathological Evaluation

Renal tissue acquisition was categorized as native kidney biopsy or image-guided biopsy of a renal mass or hilar lesion. All specimens were evaluated on the basis of light microscopy, immunofluorescence, and/or electron microscopy findings in conjunction with the clinical context and original pathology reports. Category assignment was reviewed by investigators with hematology and nephrology expertise, and overlapping features were resolved by consensus according to the dominant lesion considered most clinically relevant. With reference to previous clinicopathological studies of kidney involvement in B-cell lymphoproliferative disorders [11], and in view of the characteristics of the present cohort, dominant renal pathological categories were structurally classified as follows: (1) direct infiltrative parenchymal/interstitial pattern; (2) infiltrative pattern with concurrent glomerular/vascular lesion; (3) immune-/monoclonal protein-related glomerular pattern without definite infiltration; and (4) mass-forming renal/hilar lesion pattern. Patient-level pathological features, mixed/overlapping lesions, and renal responses are summarized in Supplementary Table S2. For glomerular lesions, attribution to the underlying B-cell neoplasm was assessed descriptively according to the presence of direct renal infiltration, monoclonal immunoglobulin-related features, immune-complex or cryoglobulinemic findings, temporal clinicopathological association, and alternative explanations such as PLA2R-associated membranous nephropathy. Cases in which causal attribution could not be established are flagged in Supplementary Table S2 as possible or uncertain/concurrent lesions.

### 2.4. Treatment and Response Assessment

Systemic anti-tumor treatment patterns were categorized according to the dominant therapeutic strategy as BTK inhibitor-based therapy, rituximab-containing non-anthracycline systemic therapy, anthracycline-containing immunochemotherapy, or supportive care only/no systemic therapy.

Hematologic response was assessed using disease-specific criteria. For CLL/SLL, response assessment was based on the iwCLL criteria [1]. For DLBCL/HGBL, response assessment was based on the Lugano classification [3].

Renal response was not defined according to a unified international cross-disease standard. Instead, it was assessed using prespecified study definitions based on renal function, 24 h urinary protein excretion, and dialysis dependence during follow-up (Supplementary Table S3). These operational renal-response definitions primarily captured functional and urinary recovery and dialysis dependence. They were not designed to fully quantify radiological regression of mass-forming renal or renal hilar lymphoma lesions; therefore, renal-response categories should not be considered entirely equivalent across glomerular/functional, directly infiltrative, and mass-forming patterns. Complete renal response (CR) was defined as recovery of serum creatinine to the institutional reference range or to the pre-involvement baseline in patients with pre-existing chronic kidney disease, together with reduction of 24 h urinary protein excretion to <0.5 g/24 h. When quantitative 24 h urinary protein data were unavailable, disappearance or near-disappearance of clinically significant proteinuria was defined as repeated negative or trace proteinuria on urinalysis. Among the six patients classified as having renal CR, five met the quantitative criterion of 24 h urinary protein excretion <0.5 g/24 h, and one relied on the qualitative urinalysis-based criterion; dialysis independence was required for patients who had required dialysis. Partial renal response (PR) was defined as a ≥30% decrease in serum creatinine or a ≥30% increase in eGFR and/or a ≥50% reduction in 24 h urinary protein excretion, without meeting criteria for CR and without progression to ESRD. No response (NR) was defined as failure to meet CR or PR criteria, and end-stage renal disease (ESRD) as progression to maintenance dialysis dependence or irreversible kidney failure. Best hematologic response and best renal response were defined as the best documented response achieved during follow-up. Follow-up duration was recorded in months and was calculated to the last available follow-up or death.

### 2.5. Statistical Analysis

Continuous variables are presented as median (range), and categorical variables as *n/N* (%). Between-group comparisons of continuous variables were performed using the Mann–Whitney U test. Fisher’s exact test was used for 2 × 2 categorical comparisons, whereas the Fisher–Freeman–Halton exact test was used for overall comparisons of multi-category variables. All tests were two-sided, and *p* < 0.05 was considered statistically significant. A total of 34 between-group comparisons were performed across Table 1, Table 2, Table 3 and Table 4. Because of the exploratory design and small sample size, no formal adjustment for multiple comparisons was performed; therefore, *p* values were interpreted descriptively, and borderline nominally significant findings were regarded as hypothesis-generating.

Given the limited sample size and the small number of outcome events, no multivariable survival regression modeling was performed, and outcome analyses were primarily descriptive. The study was not powered to detect modest differences in renal outcomes, and descriptive comparisons were interpreted cautiously. Statistical analyses were performed using IBM SPSS Statistics for Windows, version 27.0.2.0 (IBM Corp., Armonk, NY, USA), and R version 4.5.3 (R Foundation for Statistical Computing, Vienna, Austria).

A one-case sensitivity check excluding DLBCL-12, the single HGBL case with MYC and BCL2 rearrangements, was used to assess whether the principal descriptive group comparisons were materially altered.

## 3. Results

### 3.1. Baseline Characteristics at Diagnosis and Temporal Pattern of Renal Involvement Recognition

At the time of diagnosis of the underlying hematologic malignancy, there were no significant between-group differences in the proportion of patients aged ≥65 years, male sex, ECOG performance status ≥ 2, or hepatosplenomegaly. Compared with the DLBCL/HGBL group, the CLL/SLL group had higher white blood cell and absolute lymphocyte counts [15.65 (5.90–71.58) vs. 7.55 (4.60–17.80) ×10^9^/L, *p* = 0.009; 11.50 (5.10–58.40) vs. 1.15 (0.49–6.20) ×10^9^/L, *p* < 0.001]. In contrast, the DLBCL/HGBL group showed nominally higher lactate dehydrogenase and β2-microglobulin levels than the CLL/SLL group [301.00 (127.00–1177.00) vs. 188.00 (100.00–471.00) U/L, *p* = 0.011; 5.00 (2.80–8.40) vs. 3.50 (2.20–6.40) mg/L, *p* = 0.037]. No significant between-group differences were observed in hemoglobin, platelet count, albumin, 24 h urinary protein excretion, the proportion with 24 h urinary protein excretion >3.5 g/24 h, or serum creatinine. The frequencies of positive immunofixation electrophoresis and positive cryoglobulin were also comparable between the two groups.

Available disease-specific staging, prior-therapy, and molecular data are summarized in Supplementary Table S1. Ann Arbor stage and IPI score were available for all 14 DLBCL/HGBL cases, whereas Rai/Binet staging was not documented in available retrospective source records. MYC/BCL2/BCL6 FISH results were available in 8/14 DLBCL/HGBL cases; DLBCL-12 showed MYC and BCL2 rearrangements without BCL6 rearrangement, corresponding to FISH-defined MYC/BCL2 double-hit status. According to WHO HAEM5 and ICC 2022 criteria, this case was annotated as HGBL with MYC and BCL2 rearrangements, while its IHC-defined MYC/BCL2 double-expression status was reported separately. Prior systemic therapy before renal involvement recognition was recorded as yes/no/unknown in 6/6/2 CLL/SLL patients and 4/8/2 DLBCL/HGBL patients, respectively. In the one-case sensitivity check, excluding DLBCL-12 did not materially change the direction or interpretation of the principal descriptive group comparisons, including the dominant renal pathological category, tissue acquisition pattern, systemic treatment pattern, and best renal response distribution.

**Figure 2:**
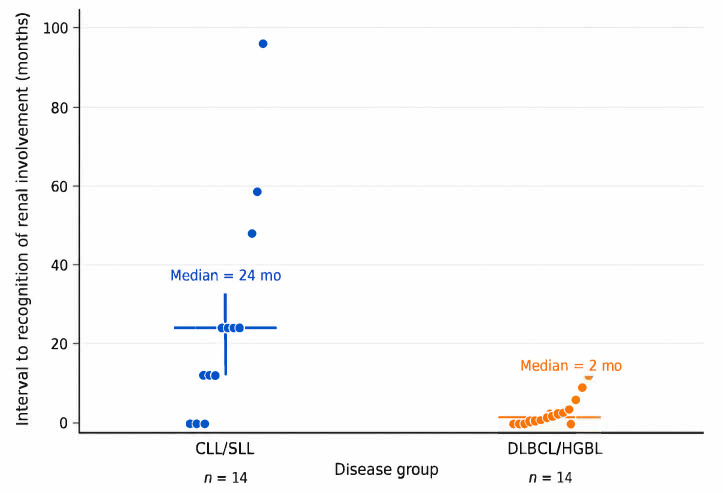
Time interval from diagnosis of hematologic malignancy to recognition of renal involvement in the CLL/SLL and DLBCL/HGBL groups. Distribution of the time interval from diagnosis of hematologic malignancy to recognition of renal involvement in the CLL/SLL and DLBCL/HGBL groups (*n* = 14 per group). Each dot represents one patient. Horizontal lines indicate the median, and vertical lines indicate the interquartile range.

The temporal pattern of renal involvement recognition differed between the two groups. The interval from diagnosis of the underlying hematologic malignancy to recognition of renal involvement was longer in the CLL/SLL group than in the DLBCL/HGBL group, with median intervals of 24.00 (0.00–96.00) months and 2.00 (0.00–12.00) months, respectively (*p* = 0.007). This interval was measured from the date of hematologic malignancy diagnosis and therefore should be interpreted in the context of the different diagnostic settings of CLL/SLL and DLBCL/HGBL. As shown in Figure 2, the CLL/SLL group had a wider distribution of time intervals, whereas most DLBCL/HGBL cases were recognized within a shorter time frame.

Renal parameters at the time of renal involvement recognition or biopsy are summarized in Table 2. Serum creatinine and eGFR at this time point were comparable between groups. However, 24 h urinary protein excretion was nominally higher in the CLL/SLL group than in the DLBCL/HGBL group [3.90 (0.65–8.20) vs. 1.58 (0.35–5.00) g/24 h, *p* = 0.039], and nephrotic syndrome or nephrotic-range proteinuria was nominally more frequent in CLL/SLL [8/14 (57.1%) vs. 2/14 (14.3%), *p* = 0.046]. In contrast, renal mass, renal hilar lesion, or obstruction on imaging was observed only in the DLBCL/HGBL group [7/14 (50.0%) vs. 0/14 (0.0%), *p* = 0.006]. Native kidney biopsy was performed in all CLL/SLL cases and in 8/14 (57.1%) DLBCL/HGBL cases, whereas image-guided renal or renal hilar lesion biopsy was performed in 6/14 (42.9%) DLBCL/HGBL cases and in no CLL/SLL cases (*p* = 0.016).

### 3.2. Dominant Clinical Entry Points and Dominant Renal Pathological Patterns

**Figure 3:**
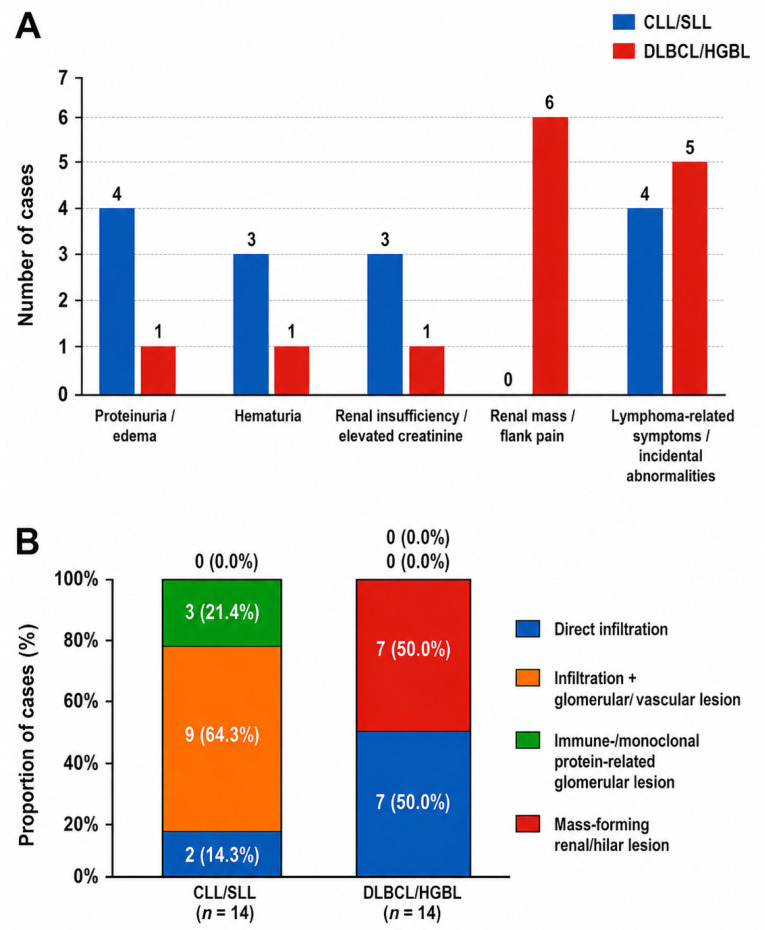
Dominant clinical entry points and dominant renal pathological categories in the CLL/SLL and DLBCL/HGBL groups. (**A**) Distribution of study-defined dominant clinical entry point categories at the time renal involvement was first recognized (*n* = 14 per group). One mutually exclusive dominant clinical entry point category was assigned to each patient. (**B**) Distribution of study-defined dominant renal pathological categories (*n* = 14 per group). Values shown on the bars indicate case counts or *n/N* (%), as appropriate.

As shown in Table 3, hematuria documented among presenting features was observed in 4/14 (28.6%) patients in each group. This variable recorded the presence of hematuria among presenting manifestations and was distinct from the mutually exclusive hematuria-predominant clinical entry point category shown in Figure 3A. Edema documented at presentation was observed only in the CLL/SLL group [3/14 (21.4%)] and in none of the DLBCL/HGBL cases, although this difference did not reach statistical significance (*p* = 0.222). Compared with the CLL/SLL group, flank pain and/or renal mass lesion at presentation was more frequent in the DLBCL/HGBL group [6/14 (42.9%) vs. 0/14 (0.0%), *p* = 0.016]. In addition, image-guided biopsy of a renal mass or hilar lesion was more common in the DLBCL/HGBL group [6/14 (42.9%) vs. 0/14 (0.0%), *p* = 0.016].

Figure 3A shows differences in the distribution of study-defined dominant clinical entry point categories between the two groups, with one mutually exclusive dominant category assigned to each patient. In the CLL/SLL group, proteinuria/edema and lymphoma-related symptoms or incidental abnormalities each accounted for 28.6% of cases, while hematuria and renal insufficiency/elevated creatinine each accounted for 21.4%; no case presented predominantly with renal mass/flank pain. In the DLBCL/HGBL group, renal mass/flank pain was the most frequent dominant clinical entry point (42.9%), followed by lymphoma-related symptoms or incidental abnormalities (35.7%); proteinuria/edema, hematuria, and renal insufficiency/elevated creatinine each accounted for 7.1% of cases.

The overall distribution of dominant renal pathological categories differed between the two groups (*p* < 0.001). In the CLL/SLL group, the most common dominant renal pathological pattern was an infiltrative pattern with concurrent glomerular/vascular lesion [9/14 (64.3%)], followed by the immune-/monoclonal protein-related glomerular pattern [3/14 (21.4%)] and the direct infiltrative parenchymal/interstitial pattern [2/14 (14.3%)]. In the DLBCL/HGBL group, the direct infiltrative parenchymal/interstitial pattern and the mass-forming renal/hilar lesion pattern each accounted for 7/14 (50.0%) cases, while no cases were assigned to the infiltrative pattern with concurrent glomerular/vascular lesion or the immune-/monoclonal protein-related glomerular pattern as the dominant category. Mixed or overlapping pathological features were identified in 10 patients, including nine CLL/SLL cases with infiltration plus glomerular/vascular or immune/monoclonal features and one DLBCL/HGBL case with direct infiltration plus a focal glomerular lesion; consensus assignment to a single dominant category was possible for all cases (Supplementary Table S2). The within-group proportions of these pathological categories are illustrated in Figure 3B.

### 3.3. Treatment Patterns and Hematologic and Renal Outcomes

The median follow-up duration was 18.5 months (range, 8–36) in the CLL/SLL group and 17.0 months (range, 7–32) in the DLBCL/HGBL group. The temporal distribution of cases according to renal involvement recognition or tissue-based diagnosis was two CLL/SLL and three DLBCL/HGBL cases in 2010–2015, five CLL/SLL and five DLBCL/HGBL cases in 2016–2020, and seven CLL/SLL and six DLBCL/HGBL cases in 2021–2025. As shown in Table 4, the overall distribution of systemic anti-tumor treatment patterns differed between the two groups (*p* < 0.001). In the CLL/SLL group, BTK inhibitor-based therapy was the most common treatment pattern [7/14 (50.0%)], followed by rituximab-containing non-anthracycline systemic therapy [6/14 (42.9%)]; one patient received supportive care only without systemic anti-tumor therapy [1/14 (7.1%)]. In the DLBCL/HGBL group, anthracycline-containing immunochemotherapy accounted for the majority of cases [13/14 (92.9%)], while one patient received BTK inhibitor-based therapy [1/14 (7.1%)].

The overall distribution of best hematologic response showed a nominal difference between the two groups (*p* = 0.043). In the CLL/SLL group, PR was the most frequent response [9/14 (64.3%)], followed by SD [3/14 (21.4%)], while CR and PD each occurred in one patient [both 7.1%]. In the DLBCL/HGBL group, CR was the most frequent best hematologic response [7/14 (50.0%)], followed by PR [4/14 (28.6%)]; PD was observed in 2/14 (14.3%) and SD in 1/14 (7.1%). In contrast, the overall distribution of best renal response did not differ significantly between the two groups (*p* = 0.922). Given the small sample size and four renal-response categories, this comparison was underpowered and should be interpreted descriptively rather than as evidence of equivalent renal outcomes. In the CLL/SLL group, PR was the most common renal response [9/14 (64.3%)], while CR and NR each accounted for 2/14 (14.3%) and ESRD for 1/14 (7.1%). In the DLBCL/HGBL group, PR was also the most common renal response [7/14 (50.0%)], followed by CR [4/14 (28.6%)], NR [2/14 (14.3%)], and ESRD [1/14 (7.1%)].

**Figure 4:**
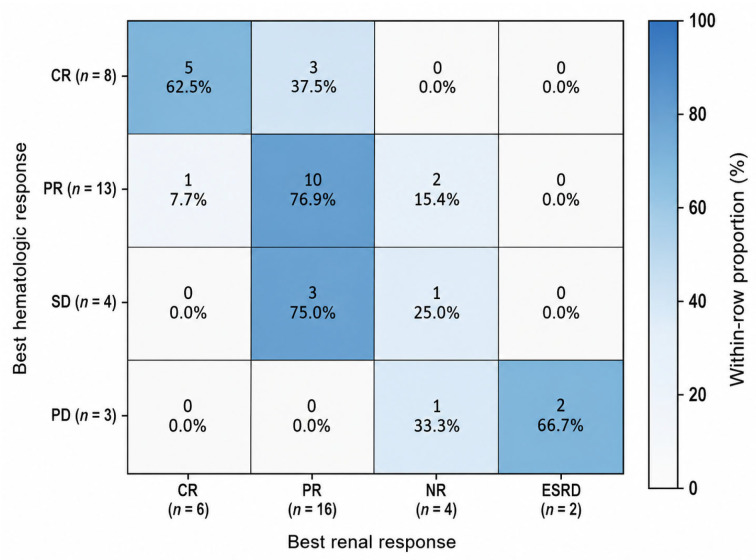
Cross-tabulation heatmap of hematologic and renal response outcomes. Heatmap showing the cross-distribution of best hematologic response and best renal response in the overall study cohort (*n* = 28). Numbers within the cells indicate case counts, and percentages indicate within-row proportions. Rows represent best hematologic response categories, and columns represent best renal response categories. This cross-tabulation is descriptive and should not be interpreted as evidence of an independent predictive relationship between hematologic and renal responses, because the renal-response construct was not equivalent across pathological patterns.

Figure 4 shows the cross-distribution of best hematologic response and best renal response in the overall cohort. Among the eight patients with hematologic CR, five had renal CR and three had renal PR. Among the 13 patients with hematologic PR, 10 had renal PR, two had renal NR, and one had renal CR. Among the four patients with hematologic SD, three had renal PR and one had renal NR. Among the three patients with hematologic PD, two had ESRD and one had NR.

Descriptive analysis by dominant pathological category suggested that renal CR/PR occurred in 8/9 patients with the direct infiltrative parenchymal/interstitial pattern, 8/9 patients with the infiltrative pattern with concurrent glomerular/vascular lesion, 5/7 patients with the mass-forming renal/hilar lesion pattern, and 1/3 patients with the immune-/monoclonal protein-related glomerular pattern without definite infiltration (Supplementary Table S2). These findings should be interpreted descriptively because of the small number of patients in each pathological category.

## 4. Discussion

In this single-center, biopsy-confirmed and tissue-selected cohort, the comparison between CLL/SLL and DLBCL/HGBL revealed observed differences at several connected levels: the hematologic background at diagnosis, the time interval to recognition of renal involvement, the dominant clinical entry point, the dominant renal pathological categories, and the distribution of hematologic and renal response outcomes. Accordingly, the present study is better understood as a descriptive comparison of observed clinicopathological patterns in patients selected through tissue diagnosis, rather than as evidence that indolent and aggressive B-cell neoplasms intrinsically involve the kidney through different biological mechanisms. Compared with existing publications that have mainly focused on single disease entities, case reports, or broader B-cell lymphoproliferative disorder cohorts, the present study adds a direct side-by-side comparison using uniform study definitions and integrates clinical entry point, tissue acquisition route, dominant renal pathological category, and response documentation within the same analytical framework.

A central consideration in interpreting these findings is that this was a biopsy-confirmed and tissue-selected cohort rather than an unselected cohort of all CLL/SLL or DLBCL/HGBL patients with renal abnormalities. The requirement for renal tissue inevitably introduced selection and ascertainment bias. In clinical practice, CLL/SLL patients are more likely to undergo native kidney biopsy when nephrological indications such as proteinuria, nephrotic syndrome, or renal dysfunction are present, whereas DLBCL/HGBL patients may undergo image-guided biopsy when a radiologically detected renal or hilar mass is identified. Therefore, the observed difference in dominant renal pathological categories partly reflects biopsy indication and tissue-acquisition route, in addition to possible disease-related clinicopathological differences.

The first point is that renal involvement did not arise in the same disease context in the two groups. Patients with CLL/SLL had higher circulating white blood cell and absolute lymphocyte counts, whereas patients with DLBCL/HGBL had higher LDH and β2-microglobulin levels; in parallel, the interval from diagnosis of the hematologic malignancy to recognition of renal involvement was substantially longer in CLL/SLL and much shorter in DLBCL/HGBL. This longer interval in the CLL/SLL group should be interpreted with caution. The interval was measured from hematologic diagnosis and is vulnerable to a lead-time effect because CLL/SLL may be diagnosed incidentally or at an asymptomatic stage years before clinically meaningful disease, whereas aggressive B-cell lymphomas such as DLBCL/HGBL are usually diagnosed at or near symptomatic presentation. Therefore, the observed difference in interval-to-recognition may reflect not only differences in clinical tempo, but also differences in when the hematologic disease was first labeled, baseline evaluation intensity, and diagnostic pathway.

We considered a sensitivity analysis anchored to the first abnormal renal parameter or first renal imaging abnormality; however, such time points were not consistently and reliably documented across the 15-year retrospective period. Accordingly, this temporal finding should be regarded as descriptive rather than as direct evidence of different intrinsic renal tropism.

Against that background, the route by which patients entered the renal diagnostic pathway also differed between groups. In CLL/SLL, renal involvement was more often first recognized through proteinuria/edema, hematuria, or renal insufficiency/elevated creatinine. In DLBCL/HGBL, renal mass/flank pain and lymphoma-related symptoms or incidental abnormalities were more prominent. This distinction is clinically relevant for hematologists. Previous work has shown that renal involvement in CLL/SLL may present with proteinuria, nephrotic syndrome, or impaired renal function, and renal insufficiency itself has been associated with adverse outcome in CLL; by contrast, kidney involvement in DLBCL/HGBL is more commonly part of aggressive extranodal disease and may first become evident during imaging or tissue work-up for a renal lesion [7,8,9,18,19,20]. In daily hematology practice, these findings support a lower threshold for considering tumor-related renal involvement when unexplained urinary abnormalities or kidney dysfunction develop in CLL/SLL, while emphasizing early imaging-guided tissue confirmation when renal mass-like lesions occur in aggressive B-cell lymphomas.

The pathological data deepen this distinction. In our cohort, CLL/SLL was mainly associated with an infiltrative pattern accompanied by glomerular or vascular lesions, together with a smaller but relevant proportion of immune-/monoclonal protein-related glomerular lesions. DLBCL/HGBL, in contrast, was dominated by direct infiltrative parenchymal/interstitial lesions and mass-forming renal or hilar lesions [10,11]. These findings closely parallel the clinicopathological literature on B-cell lymphoproliferative disorders involving the kidney. Studies dedicated to CLL/SLL have emphasized its broad renal pathological spectrum, whereas biopsy-proven cohorts of B-cell lymphomas have shown that aggressive entities are enriched for direct malignant infiltration, acute kidney injury, and mass-forming disease [21,22]. Taken together, the observed pathological differences are clinically useful descriptive patterns, but they should be interpreted in the context of tissue selection and biopsy route rather than as definitive proof of intrinsic disease-specific renal tropism.

The difference in tissue acquisition represents an important source of potential selection bias. All CLL/SLL cases underwent native kidney biopsy, whereas a subset of DLBCL/HGBL cases underwent image-guided biopsy of renal mass or hilar lesions. Therefore, the higher frequency of mass-forming lesions in DLBCL/HGBL may partly reflect the diagnostic pathway and biopsy approach. However, this pattern also mirrors real-world clinical practice, in which DLBCL/HGBL more often enters the renal diagnostic pathway through imaging-detected lesions, while CLL/SLL more often presents with urinary abnormalities or renal dysfunction. This point is particularly important because tissue-acquisition route was closely related to the dominant pathological category in this cohort.

This difference in pathological expression also explains why tissue diagnosis is central to management. Broader onco-nephrology reviews have emphasized that kidney involvement in hematologic malignancies may result from several non-mutually exclusive mechanisms, including direct tumor infiltration, immune-mediated glomerular injury, monoclonal protein-related lesions, obstruction, and treatment-related toxicity [4,23]. Earlier literature on CLL-associated nephrotic syndrome likewise supports the view that glomerular involvement in CLL may be immune-complex-mediated or paraneoplastic rather than attributable to one stereotyped lesion [24,25]. In this setting, clinical and laboratory data alone are rarely sufficient to define the dominant process. The biopsy pattern in our study—predominantly native kidney biopsy in CLL/SLL and more frequent image-guided sampling of renal masses or hilar lesions in DLBCL/HGBL—therefore reflects not only different anatomical presentations but also different diagnostic decisions made in routine practice.

The attribution of glomerular lesions should be interpreted cautiously. Renal involvement in B-cell neoplasms may include biologically distinct processes, including direct infiltration, immune-complex or paraneoplastic glomerular injury, monoclonal immunoglobulin-related lesions, cryoglobulinemic glomerulonephritis, thrombotic microangiopathy, and coincidental primary glomerular disease. In particular, the PLA2R-associated membranous nephropathy case was flagged as an uncertain/concurrent lesion rather than as definitively attributable to CLL/SLL, and membranous nephropathy cases in which secondary membranous nephropathy could not be excluded were considered possible but not definite lymphoma-related lesions. Therefore, the observed pathological heterogeneity in CLL/SLL should be interpreted as a descriptive finding in this tissue-selected cohort rather than as evidence that all glomerular lesions were causally produced by CLL/SLL.

The treatment and outcome data further reinforce the need for descriptive interpretation. The marked difference in systemic anti-tumor treatment patterns was expected, given disease-specific standards of care for CLL/SLL and DLBCL/HGBL. The nominal difference in best hematologic response should be interpreted cautiously in view of multiple comparisons, and the absence of a statistically significant difference in renal-response distribution should not be interpreted as evidence that renal outcomes were truly comparable between groups. With only 14 patients per group and four renal-response categories, this analysis was underpowered and should be regarded as descriptive. Figure 4 shows the cross-distribution of hematologic and renal responses; however, this heatmap should not be interpreted as evidence of an independent predictive relationship. In DLBCL/HGBL cases with mass-forming disease, renal recovery may partly track systemic lymphoma response, making hematologic–renal concordance partly built into the response framework. Descriptive analysis by pathological category suggested that renal recovery may be less frequent in immune-/monoclonal protein-related glomerular lesions without definite infiltration than in direct infiltrative or mass-forming patterns, but the number of patients in each category was too small for formal prognostic inference.

Because no unified international renal response standard applies across CLL/SLL and DLBCL/HGBL with heterogeneous renal involvement, renal responses in this study were assessed using prespecified operational definitions based on renal function, proteinuria, and dialysis dependence. These definitions approximate commonly used nephrology concepts such as complete or partial remission of proteinuric disease and recovery or stabilization of renal function, but they cannot be fully harmonized with disease-specific nephrology trial endpoints. Creatinine, eGFR, proteinuria, and dialysis dependence are clinically meaningful readouts for glomerular or functional renal involvement, but they do not fully capture radiological regression of mass-forming renal or renal hilar lymphoma lesions. Therefore, renal-response categories should not be considered entirely equivalent across glomerular/functional, directly infiltrative, and mass-forming patterns.

Several limitations should be acknowledged. This was a retrospective single-center study with a small sample size, and the conclusions mainly describe biopsy-confirmed patterns rather than providing a basis for formal prognostic modeling. The study was not powered to detect modest differences in renal outcomes. In addition, the dominant presenting manifestation categories and dominant renal pathological categories were structured, study-defined classifications created for comparative analysis, and renal response was assessed using prespecified study criteria rather than a unified cross-disease international standard. Multiplicity should also be considered when interpreting the between-group comparisons. Although several variables reached nominal statistical significance, the analyses involved 34 comparisons across Table 1, Table 2, Table 3 and Table 4 without formal adjustment for multiple testing. Therefore, borderline findings, including β2-microglobulin, proteinuria at renal involvement recognition, nephrotic syndrome/nephrotic-range proteinuria, and best hematologic response, should be interpreted cautiously as hypothesis-generating rather than confirmatory. Best-response outcomes should be interpreted in the context of the available follow-up duration, because the probability of documenting a best hematologic or renal response depends on the observation window. Formal survival or renal-survival analyses were not performed because of the small sample size. Treatment-pattern and response comparisons should also be interpreted in the context of calendar time. Although both disease groups included cases across the three study periods, the overall inclusion window spanned 2010–2025, during which CLL/SLL management evolved from chemoimmunotherapy-based approaches toward BTK-inhibitor-based therapy. Therefore, the observed treatment pattern difference reflects both disease-specific therapeutic standards and treatment-era effects. Disease-specific molecular and prognostic variables were not uniformly available because of the long retrospective study period. Ann Arbor stage and IPI score were available for all DLBCL/HGBL cases, whereas Rai/Binet staging was not documented in the available retrospective source records, and MYC/BCL2/BCL6 FISH testing was available in only a subset of DLBCL/HGBL patients. The DLBCL/HGBL cohort should therefore be interpreted as clinically and molecularly heterogeneous, particularly because one case showed MYC/BCL2 double-hit features without BCL6 rearrangement. Even so, the present study adds value by placing renal involvement in CLL/SLL and DLBCL/HGBL within the same clinical hematology framework. Overall, our data indicate observed descriptive differences in the tempo, clinical entry point, and pathological expression of renal involvement between these two tissue-selected groups. In practice, unexplained urinary abnormalities or renal dysfunction in CLL/SLL should prompt timely consideration of kidney biopsy when clinically feasible, whereas renal mass-forming lesions or rapid renal deterioration in DLBCL/HGBL should be integrated early into staging, tissue diagnosis, and treatment planning. Larger multicenter studies with pathology-stratified analyses are needed to determine how these patterns relate to long-term renal and hematologic outcomes [26,27]. DLBCL-12 was retained in the aggressive comparator group but explicitly annotated as HGBL with MYC and BCL2 rearrangements; a sensitivity check excluding this case did not materially change the direction or interpretation of the principal descriptive comparisons. In addition, recent translational studies of B-cell non-Hodgkin lymphoma have highlighted the clinical and biological heterogeneity of this disease spectrum, supporting future integration of clinicopathological and molecular perspectives in studies of organ involvement [28].

## 5. Conclusions

In this single-center, retrospective, biopsy-confirmed and tissue-selected cohort, CLL/SLL and DLBCL/HGBL showed different observed patterns of renal involvement recognition, tissue acquisition, dominant renal pathological categories, treatment approaches, and response documentation. These differences should be interpreted as descriptive clinicopathological patterns rather than as definitive evidence that indolent and aggressive B-cell neoplasms intrinsically involve the kidney through different biological mechanisms. Selection and ascertainment bias, biopsy-route effects, potential lead-time bias, incomplete disease-specific characterization, limited sample size, and the non-equivalence of renal-response constructs across pathological patterns should be considered when interpreting the findings. Despite these limitations, the study supports careful tissue-based evaluation of renal abnormalities in B-cell neoplasms and provides hypothesis-generating data for future multicenter studies.

## Figures and Tables

**Table 1:** Baseline demographic and clinical characteristics of patients with renal involvement in the CLL/SLL and DLBCL/HGBL groups.

Variable	CLL/SLL (*n* = 14)	DLBCL/HGBL (*n* = 14)	*p* Value
Age ≥ 65 years	8/14 (57.1)	4/14 (28.6)	0.252
Male sex	9/14 (64.3)	5/14 (35.7)	0.257
ECOG performance status ≥ 2	6/14 (42.9)	7/14 (50.0)	1
Interval from hematologic malignancy diagnosis to renal involvement (months)	24.00 (0.00–96.00)	2.00 (0.00–12.00)	0.007
Hepatosplenomegaly *	4/14 (28.6)	6/14 (42.9)	0.695
WBC, ×10^9^/L	15.65 (5.90–71.58)	7.55 (4.60–17.80)	0.009
ALC, ×10^9^/L	11.50 (5.10–58.40)	1.15 (0.49–6.20)	<0.001
Hemoglobin, g/L	99.50 (79.00–177.00)	102.00 (64.00–126.00)	0.818
Platelet count, ×10^9^/L	128.50 (57.00–221.00)	164.00 (79.00–368.00)	0.241
LDH, U/L	188.00 (100.00–471.00)	301.00 (127.00–1177.00)	0.011
β2-microglobulin, mg/L	3.50 (2.20–6.40)	5.00 (2.80–8.40)	0.037
Albumin, g/L	35.25 (18.70–46.70)	34.60 (20.10–47.70)	0.963
Positive immunofixation electrophoresis, *n*/tested †	3/14 (21.4)	0/11 (0.0)	0.23
Positive cryoglobulin, *n*/tested ‡	4/14 (28.6)	1/11 (9.1)	0.341
24 h urinary protein excretion, g/24 h	3.40 (0.41–8.16)	1.50 (0.21–4.86)	0.063
24 h urinary protein excretion > 3.5 g/24 h	7/14 (50.0)	3/14 (21.4)	0.236
Serum creatinine, μmol/L	131.18 (50.80–503.00)	187.00 (85.40–396.00)	0.395

Notes: Continuous variables are presented as median (range), and categorical variables as *n/N* (%). Continuous variables were compared using the Mann–Whitney U test, and categorical variables were compared using Fisher’s exact test. The variable “interval from hematologic malignancy diagnosis to renal involvement (months)” refers to the time from diagnosis of the primary hematologic malignancy to recognition of renal involvement. Renal parameters in Table 1 were recorded at the time of hematologic malignancy diagnosis and should not be interpreted as renal-recognition or biopsy-proximate values. * Hepatosplenomegaly included splenomegaly, hepatomegaly, or hepatosplenomegaly. † Positive immunofixation electrophoresis is presented as *n*/tested; three patients in the DLBCL/HGBL group were not tested. ‡ Positive cryoglobulin is presented as *n*/tested; three patients in the DLBCL/HGBL group were not tested. Abbreviations: CLL, chronic lymphocytic leukemia; SLL, small lymphocytic lymphoma; DLBCL, diffuse large B-cell lymphoma; ECOG, Eastern Cooperative Oncology Group; WBC, white blood cell count; ALC, absolute lymphocyte count; LDH, lactate dehydrogenase; HGBL, high-grade B-cell lymphoma.

**Table 2:** Renal status at recognition of renal involvement or biopsy in the CLL/SLL and DLBCL/HGBL groups.

Variable	CLL/SLL (*n* = 14)	DLBCL/HGBL (*n* = 14)	*p* Value
Serum creatinine, μmol/L	280.50 (76.00–690.00)	246.00 (96.00–710.00)	0.854
eGFR, mL/min/1.73 m^2^	18.85 (5.50–77.30)	20.00 (5.90–77.10)	0.982
24 h urinary protein, g/24 h	3.90 (0.65–8.20)	1.58 (0.35–5.00)	0.039
Hematuria	10/14 (71.4)	10/14 (71.4)	1.000
Nephrotic syndrome or nephrotic-range proteinuria	8/14 (57.1)	2/14 (14.3)	0.046
Dialysis requirement	4/14 (28.6)	4/14 (28.6)	1.000
Renal mass, renal hilar lesion, or obstruction on imaging	0/14 (0.0)	7/14 (50.0)	0.006
Native kidney biopsy	14/14 (100.0)	8/14 (57.1)	0.016
Image-guided renal/renal hilar lesion biopsy	0/14 (0.0)	6/14 (42.9)	0.016

Notes: Continuous variables are presented as median (range), and categorical variables as *n/N* (%). Values were obtained from the closest available records before renal biopsy or tissue-based diagnosis; when biopsy-proximate data were unavailable, records at renal involvement recognition were used. Hematuria was based on urinalysis or clinical records and is not identical to hematuria as the dominant clinical entry point. Nephrotic-range proteinuria was defined as 24 h urinary protein excretion > 3.5 g/24 h. Imaging-detected renal mass, hilar lesion, or obstruction was recorded at renal involvement recognition or biopsy and is not identical to renal mass/flank pain as the dominant clinical entry point or to image-guided biopsy. Abbreviations: CLL, chronic lymphocytic leukemia; SLL, small lymphocytic lymphoma; DLBCL, diffuse large B-cell lymphoma; eGFR, estimated glomerular filtration rate; HGBL, high-grade B-cell lymphoma.

**Table 3:** Renal presentations, tissue acquisition, and dominant renal pathological categories in the CLL/SLL and DLBCL/HGBL groups.

Variable	CLL/SLL (*n* = 14)	DLBCL/HGBL (*n* = 14)	*p* Value
**Renal presentations and tissue acquisition**			
Hematuria at presentation	4/14 (28.6)	4/14 (28.6)	1
Edema at presentation	3/14 (21.4)	0/14 (0.0)	0.222
Flank pain and/or renal mass lesion at presentation	0/14 (0.0)	6/14 (42.9)	0.016
Image-guided biopsy of renal mass or hilar lesion	0/14 (0.0)	6/14 (42.9)	0.016
**Dominant renal pathological category**			<0.001
Direct infiltrative parenchymal/interstitial pattern	2/14 (14.3)	7/14 (50.0)	
Infiltrative pattern with concurrent glomerular/vascular lesion	9/14 (64.3)	0/14 (0.0)	
Immune-/monoclonal protein-related glomerular pattern without definite infiltration	3/14 (21.4)	0/14 (0.0)	
Mass-forming renal/hilar lesion pattern	0/14 (0.0)	7/14 (50.0)	

Notes: Categorical variables are presented as *n/N* (%). Between-group comparisons for renal presentations and tissue acquisition were performed using Fisher’s exact test. The overall distribution of dominant renal pathological categories was compared using the Fisher–Freeman–Halton exact test. Variables in the “renal presentations and tissue acquisition” section indicate selected presenting features or biopsy approaches recorded in the medical records and are not mutually exclusive dominant clinical entry point categories. “Hematuria at presentation” indicates documented hematuria among presenting features, whereas the hematuria-predominant clinical entry point category indicates that hematuria was judged to be the principal feature leading to recognition of renal involvement. “Image-guided biopsy of renal mass or hilar lesion” refers to targeted tissue acquisition from a renal mass or hilar lesion; all remaining cases underwent native kidney biopsy. Dominant renal pathological categories were assigned according to the principal lesion pattern identified on renal tissue pathology.

**Table 4:** Systemic anti-tumor treatment patterns and hematologic and renal response outcomes in the CLL/SLL and DLBCL/HGBL groups.

Variable	CLL/SLL (*n* = 14)	DLBCL/HGBL (*n* = 14)	*p* Value
**Systemic anti-tumor treatment pattern**			<0.001
BTK inhibitor-based therapy	7/14 (50.0)	1/14 (7.1)	
Rituximab-containing non-anthracycline systemic therapy	6/14 (42.9)	0/14 (0.0)	
Anthracycline-containing immunochemotherapy	0/14 (0.0)	13/14 (92.9)	
Supportive care only/no systemic therapy	1/14 (7.1)	0/14 (0.0)	
**Best hematologic response**			0.043
CR	1/14 (7.1)	7/14 (50.0)	
PR	9/14 (64.3)	4/14 (28.6)	
SD	3/14 (21.4)	1/14 (7.1)	
PD	1/14 (7.1)	2/14 (14.3)	
**Best renal response**			0.922
CR	2/14 (14.3)	4/14 (28.6)	
PR	9/14 (64.3)	7/14 (50.0)	
NR	2/14 (14.3)	2/14 (14.3)	
ESRD	1/14 (7.1)	1/14 (7.1)	

Notes: Categorical variables are presented as *n/N* (%). The overall distributions of systemic anti-tumor treatment patterns, best hematologic response, and best renal response were compared using the Fisher–Freeman–Halton exact test. Treatment patterns were categorized according to the dominant therapeutic strategy. NR, no response; ESRD, end-stage renal disease.

## Data Availability

The data presented in this study are available from the corresponding author upon reasonable request, subject to institutional and ethical restrictions.

## Supplementary Materials

The following supporting information can be downloaded at https://www.mdpi.com/article/10.3390/curroncol33070420/s1: Supplementary Table S1. Disease-specific characteristics available in the retrospective records; Supplementary Table S2. Pathological features, dominant renal pathological categories, and renal responses; Supplementary Table S3. Study-defined renal response criteria; Supplementary Table S4. Disease-group breakdown of excluded patients.
